# Ortner’s syndrome: a case report and literature review*

**DOI:** 10.1590/0100-3984.2013.1836

**Published:** 2015

**Authors:** Bruno Landim Dutra, Lenilton da Costa Campos, Hélder de Castro Marques, Vagner Moysés Vilela, Rodolfo Elias Diniz da Silva Carvalho, André Geraldo da Silva Duque

**Affiliations:** 1 MD, Resident, Unit of Radiology and Imaging Diagnosis, Hospital Universitário – Universidade Federal de Juiz de Fora (HU-UFJF), Juiz de Fora, MG, Brazil.; 2 MDs, Radiologists, Unit of Radiology and Imaging Diagnosis, Hospital Universitário – Universidade Federal de Juiz de Fora (HU-UFJF) and Grupo Alliar, Juiz de Fora, MG, Brazil.; 3 MD, Radiologist, Unit of Radiology and Imaging Diagnosis, Hospital Universitário – Universidade Federal de Juiz de Fora (HU-UFJF), Juiz de Fora, MG, Brazil.; 4 MD, Radiologist at CDM Imagem – Centro de Diagnóstico Médico, and Cedim – Centro Diagnóstico em Medicina, São Mateus, ES, Brazil.; 5 MD, Radiologist at Axial Medicina Diagnóstica, Belo Horizonte, MG, Brazil.

**Keywords:** Ortner’s syndrome, Aortic arch aneurysm, Recurrent laryngeal nerve, Dysphonia, Dry cough

## Abstract

The authors report the case of a 55-year-old female, hypertensive, smoker patient presenting with dysphonia, dysphagia and persistent dry cough. Laryngoscopy diagnosed left vocal cord paralysis. Computed tomography demonstrated saccular aneurysm of the inferior wall of the aortic arch, stretching the left recurrent laryngeal nerve, a finding compatible with Ortner’s syndrome.

## Abstract (pt)

Relatamos um caso de paciente do sexo feminino, 55 anos, hipertensa, tabagista, apresentando disfonia, disfagia e tosse seca persistente. À videolaringoscopia foi observada paralisia da prega vocal esquerda. A tomografia computadorizada mostrou aneurisma sacular da parede inferior do arco aórtico, no local de deflexão do nervo laríngeo recorrente, permitindo diagnosticar a síndrome de Ortner.

## INTRODUCTION

In 1897, Ortner described the association between mitral stenosis and hoarseness related to paralysis of the left recurrent laryngeal nerve^(1)^. Ortner’s syndrome, whose physiopathology is related to compromise of the recurrent laryngeal nerve between the aorta and the pulmonary artery, may be present in other diseases of benign cardiovascular origin^(1)^.

In the present report, the authors describe the case of a patient presenting with symptoms compatible with the syndrome as a result of a saccular aneurysm located in the inferior wall of the aortic arch, determining an injury to the mentioned nerve.

## CASE REPORT

**Figure 1:**
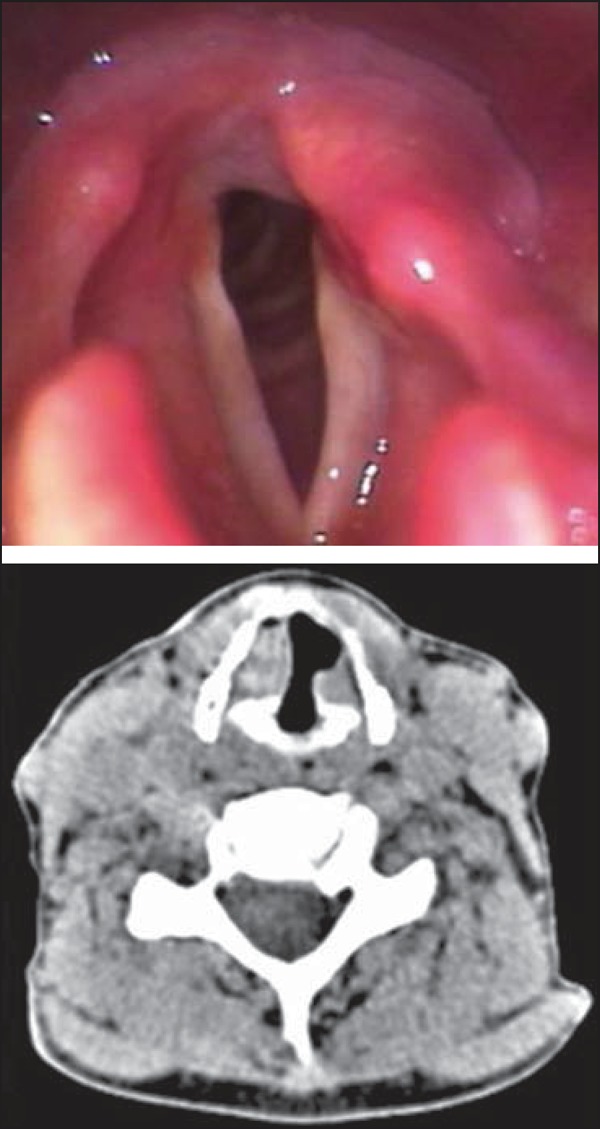
Documentation of videolaryngoscopy and axial CT section (soft tissue window) demonstrating vocal cords asymmetry, with signs of paralysis at left.

A 55-year-old, female, hypertensive, smoker (35 pack year) patient presenting with dysphonia, mild dysphagia and persistent dry cough for four months. Videolaryngoscopy demonstrated paramedian left vocal cord paralysis (Figure 1).

**Figure 2:**
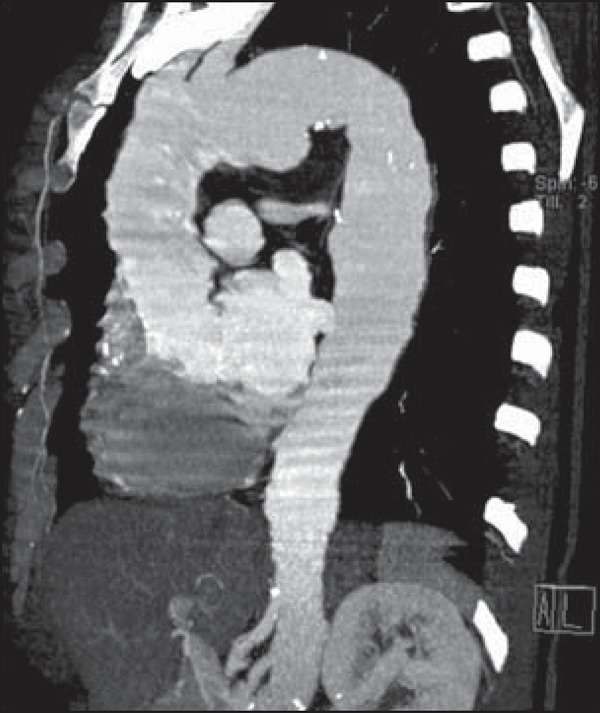
Sagittal oblique reconstruction with the maximum intensity projection technique identifying the aneurysm on the inferior wall of the aortic arch where the recurrent laryngeal nerve usually travels along this vessel.

**Figure 3:**
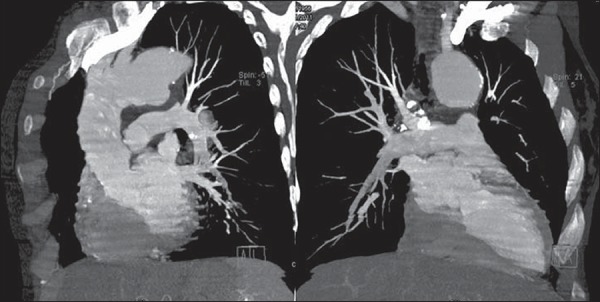
Maximum intensity projection, multiplanar reformation obtaining oblique images demonstrating the anatomical relationship between the aortic arch, pulmonary artery trunk and the aneurysm projected on the aortopulmonary window.

**Figure 4:**
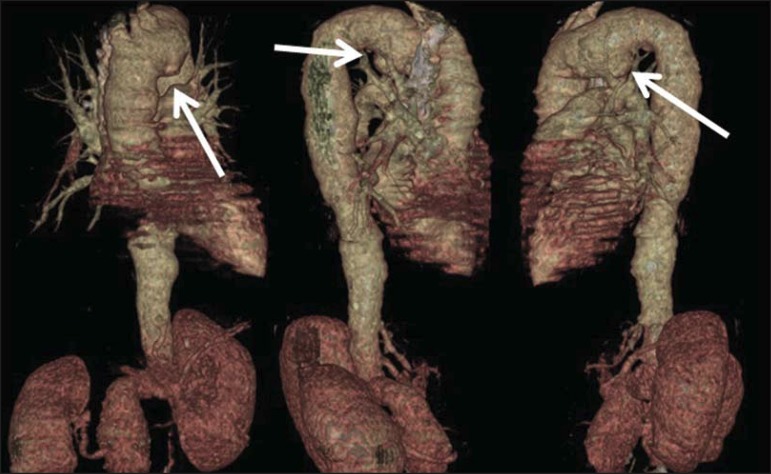
Right and left, anteroposterior and oblique multiplanar reconstructions, utilizing the volume rendering technique, demonstrating focal dilatation of the inferior wall of the aortic arch (arrows).

Computed tomography of cervical and thoracic regions confirmed the videolaryngoscopy findings (Figure 1) and defined the diagnosis by demonstrating the presence of a focal, saccular aneurysm on the inferior wall of the aortic arch (Figures 2, 3 and 4), responsible for stretching the left recurrent laryngeal nerve and causing the described symptoms.

## DISCUSSION

Persistent cough, dysphagia and dysphonia may suggest vocal cord paralysis secondary to injury of the recurrent laryngeal nerve. The vocal cords receive motor innervation by a vagus nerve branch, the recurrent laryngeal nerve. At right, the recurrent laryngeal nerve travels along the inferior surface of the subclavian artery and, at left, it travels inferiorly to the aortic arch in the aortopulmonary window, upwards to the larynx, adjacent to the tracheoesophageal groove. The symptoms may be determined by any compression/stretching of the vagus nerve, at its origin in the lateral sulcus posterior to the bulb, on its course in the bulbocerebellar cistern, at the point where it emerges from the skull through the jugular foramen, in the carotid sheath or adjacent to the sites of recurrent laryngeal nerves deflection in the above mentioned vessels^(2)^.

Ortner’s syndrome, also known as cardiovocal syndrome, results from compression of the recurrent laryngeal nerve secondary to benign cardiovascular causes (Table 1)^(1-3)^. Other conditions may affect the mentioned nerve, however without causing the syndrome^(2,4,5)^.

**Table 1:** Differential diagnosis of medial causes of involvement of recurrent laryngeal nerve.

Vascular/cardiac	Aortic dissection or pseudoaneurysm, left atrium enlargement, congenital heart diseases, pulmonary artery enlargement, pulmonary embolism
Neoplastic	Bronchogenic carcinoma, lymphoma, esophageal carcinoma, neurogenic tumors (paraganglioma, schwannoma), thyroid carcinoma, malignant thymus disease, lymph node metastasis; retrosternal goiter
Surgical/iatrogenic	Heart surgery, median sternotomy, patent ductus arteriosus ligation or embolization, left lobectomy/pneumomectomy, mediastinoscopy, radical esophagectomy, tracheal resection, thymectomy, thyroidectomy, anterior approach in spine surgeries, carotid endarterectomy, external radiotherapy
Inflammatory	Sarcoidosis, silicosis, fibrosing mediastinitis
Infiltrative	Amyloidosis
Infectious	Tuberculosis, histoplasmosis, coccidioidomycosis, bacterial abscess, mycotic aortic pseudoaneurysm
Traumatic	Deceleration injuries, penetrating chest injuries

The tomographic findings of vocal cord paralysis include: thickening and medial positioning of the aryepiglottic fold; increased volume of the piriform sinus and ipsilateral ventricles; anteromedial positioning of the arytenoids cartilage; paramedian position of the vocal cord. In the coronal plane, one may observe subglottic arch flattening^(2)^.

The diagnostic confirmation includes endoscopic studies of the larynx and skull, neck and chest computed tomography. The tomographic evaluation is aimed at demonstrating, besides the laryngeal abnormalities, the presence of possible lesions in the course of the vagus and recurrent laryngeal nerves, from the skull base to the aortopulmonary window^(3,6,7)^, corroborating the diagnosis.
